# Exchange Transfusion Simulation Models: A Technical Report

**DOI:** 10.7759/cureus.5317

**Published:** 2019-08-04

**Authors:** Orna Rosen, Robert Angert

**Affiliations:** 1 Neonatology, Pediatrics, Children's Hospital at Montefiore, Montefiore Medical Center, New York, USA; 2 Neonatology, New York University Langone Health, New York, USA

**Keywords:** umbilical venous catheter, hyperbilirubinemia, simulation, umbilical artery catheter, exchange transfusion, abo incompatibility

## Abstract

This technical report describes the creation of two exchange transfusion models in the newborn. These are low cost, easy to assemble, authentic, and realistic task trainers that provide the opportunity for neonatal providers to practice this rare, life-saving procedure. A critical action checklist is included to guide the simulated procedure. Also included is a suggested clinical scenario with optional complications and variations.

## Introduction

Double volume exchange (DVE) transfusion used to be a common procedure, first performed in 1925 by Hart [1]. New treatments, such as Rho D immune globulin for anti-D hemolytic disease, phototherapy, and intravenous immunoglobulin (IVIG), have decreased the need for DVE transfusion [2], and it has since become a rare procedure with many providers lacking the necessary skills and experience. DVE transfusion is a risky procedure that can result in complications like arrhythmias, cardiac arrest, air embolus, metabolic derangements like hypocalcemia and hypoglycemia, hematological complications, such as thrombocytopenia, as well as an infection. Providers in neonatology need to master this life-saving procedure. In our Neonatal Intensive Care Units, we teach DVE transfusion using simulation to our neonatal fellows, nurse practitioners, and physician assistants every few months. A simple, inexpensive task trainer was created for this purpose, and instructions for assembling it are described in this report.

## Technical report

Exchange transfusion task trainer, low cost, realistic, easy to use

Exchange Model

A life-sized fabric doll ($9.99) (Ikea, Leiden, Netherlands) was modified for this procedure. The supplies used were a zipper, an infant suction bulb ($2.99), a reusable rubber umbilical stump ($20) (Laerdal Medical, Stavanger, Norway), and an expired exchange transfusion kit. A bag of normal saline, cornstarch, and red, green, and blue food coloring were used to simulate the color and viscosity of blood. 

**Figure 1:**
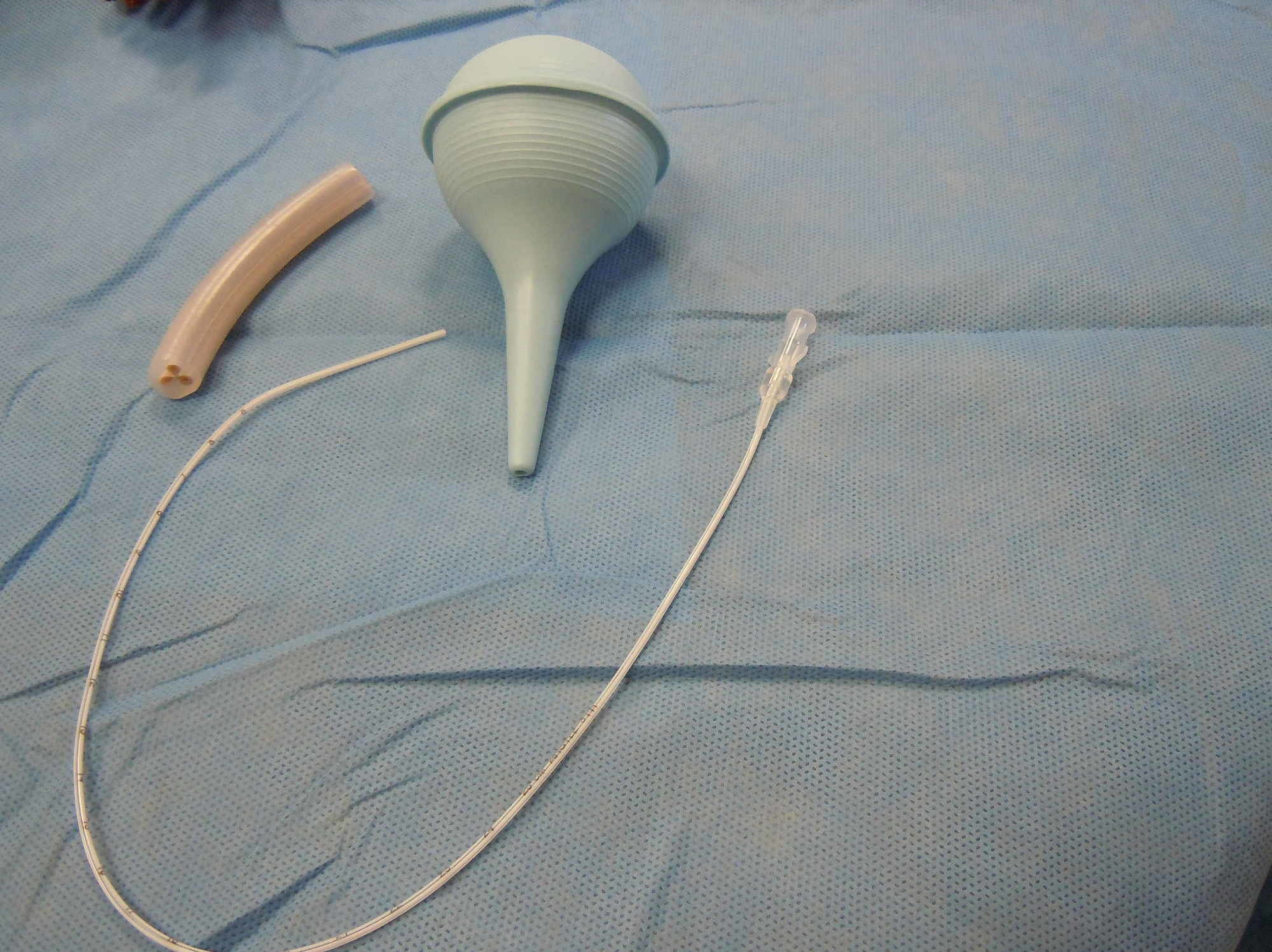
Materials for constructing the reservoir

**Figure 2:**
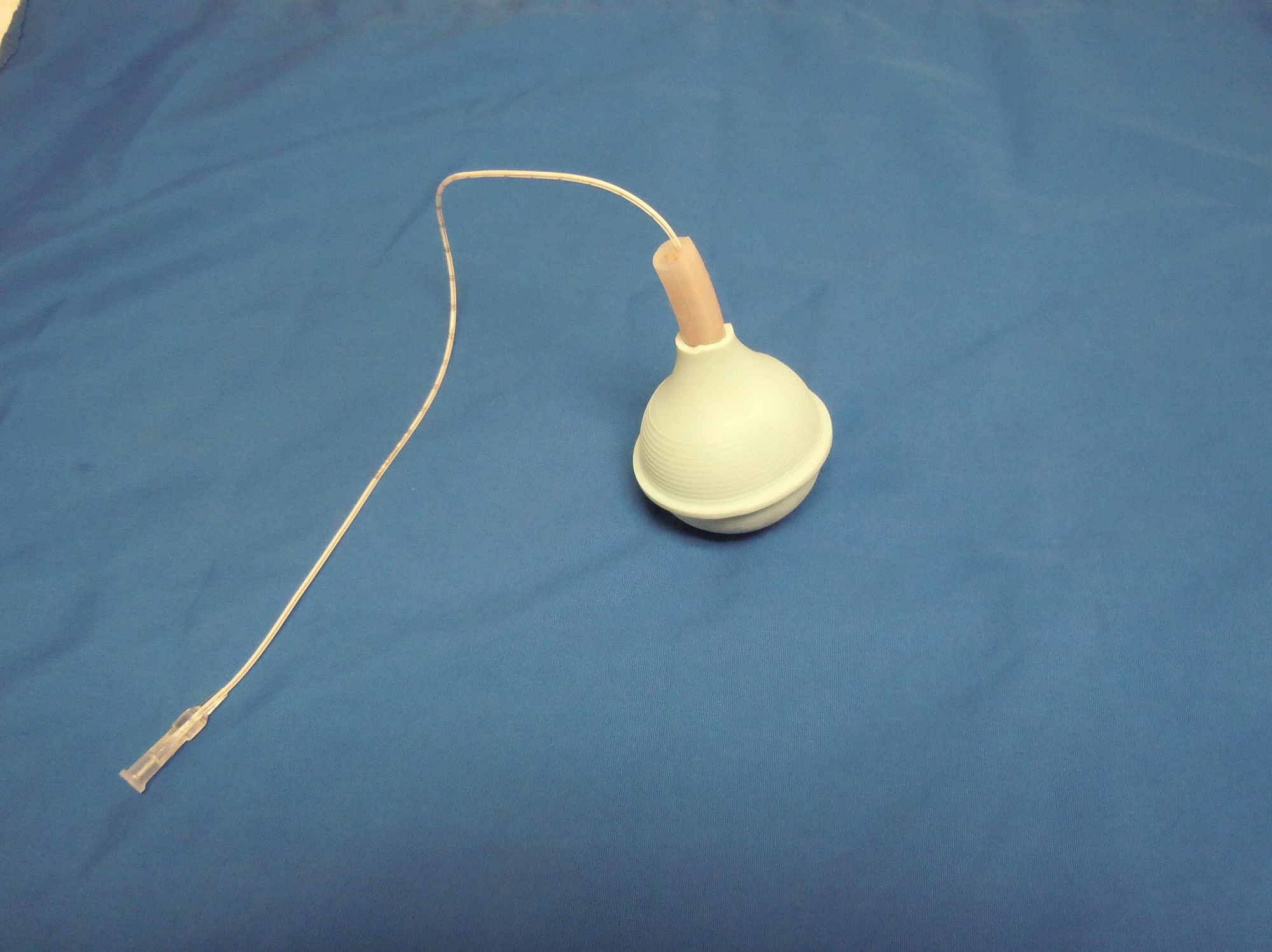
Fully assembled reservoir

The suction bulb is cut one inch from the tip so that the rubber umbilical stump can be inserted in the end. The bulb serves as a reservoir for the simulated blood (Figures 1-2).

**Figure 3:**
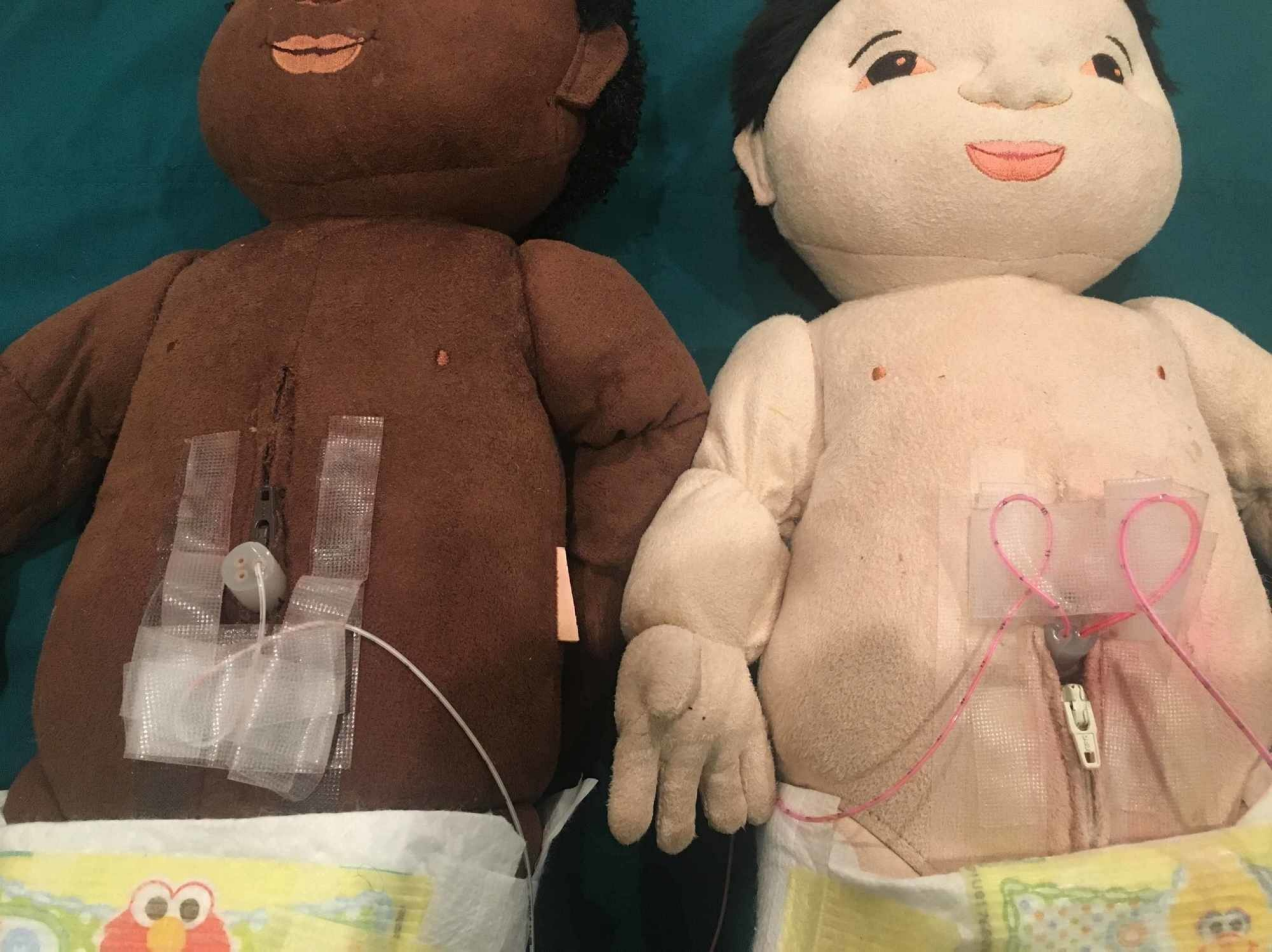
Model with zipper above and below simulated umbilicus

In the model, we cut a segment on the doll’s abdomen 3.5" to 4" either above or below the location of the umbilicus. The suction bulb and umbilical stump are inserted into this cavity. A zipper is sewn into the model either above or below the umbilical stump as shown in the accompanying image (Figure 3). 

**Figure 4:**
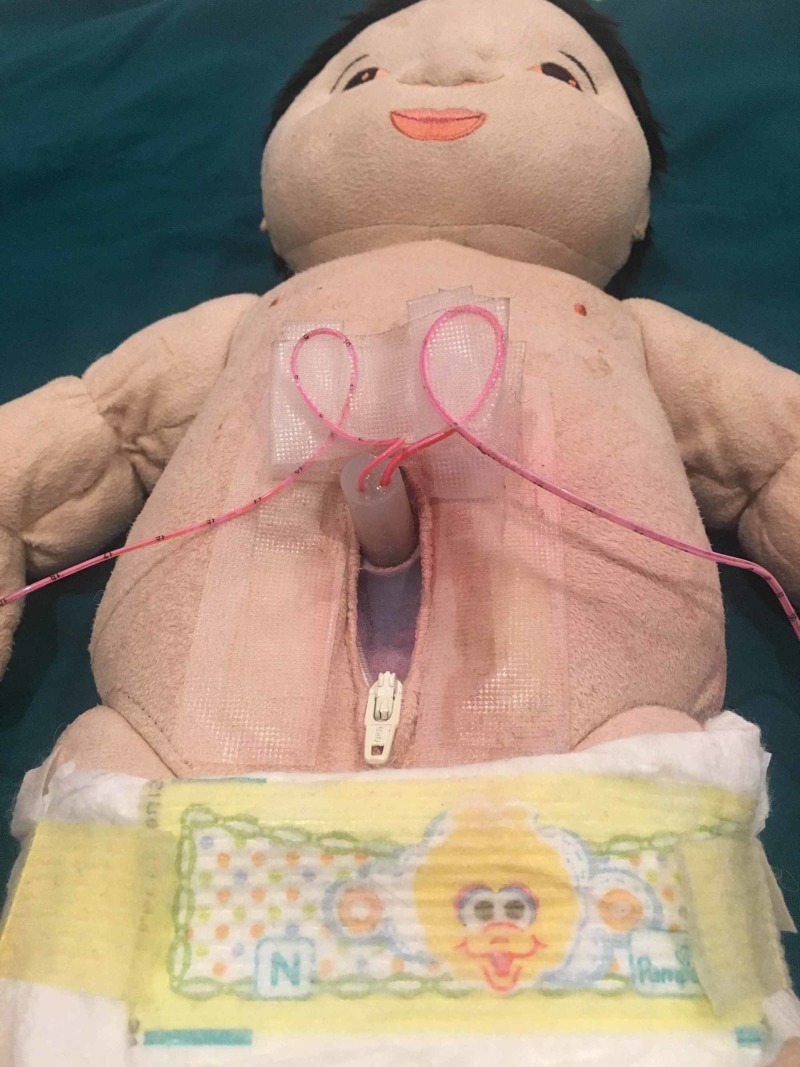
Model with zipper open for removal and cleaning reservoir

The advantage of using the zipper is that the reservoir may be removed for cleaning or replacement (Figure 4). 

**Figure 5:**
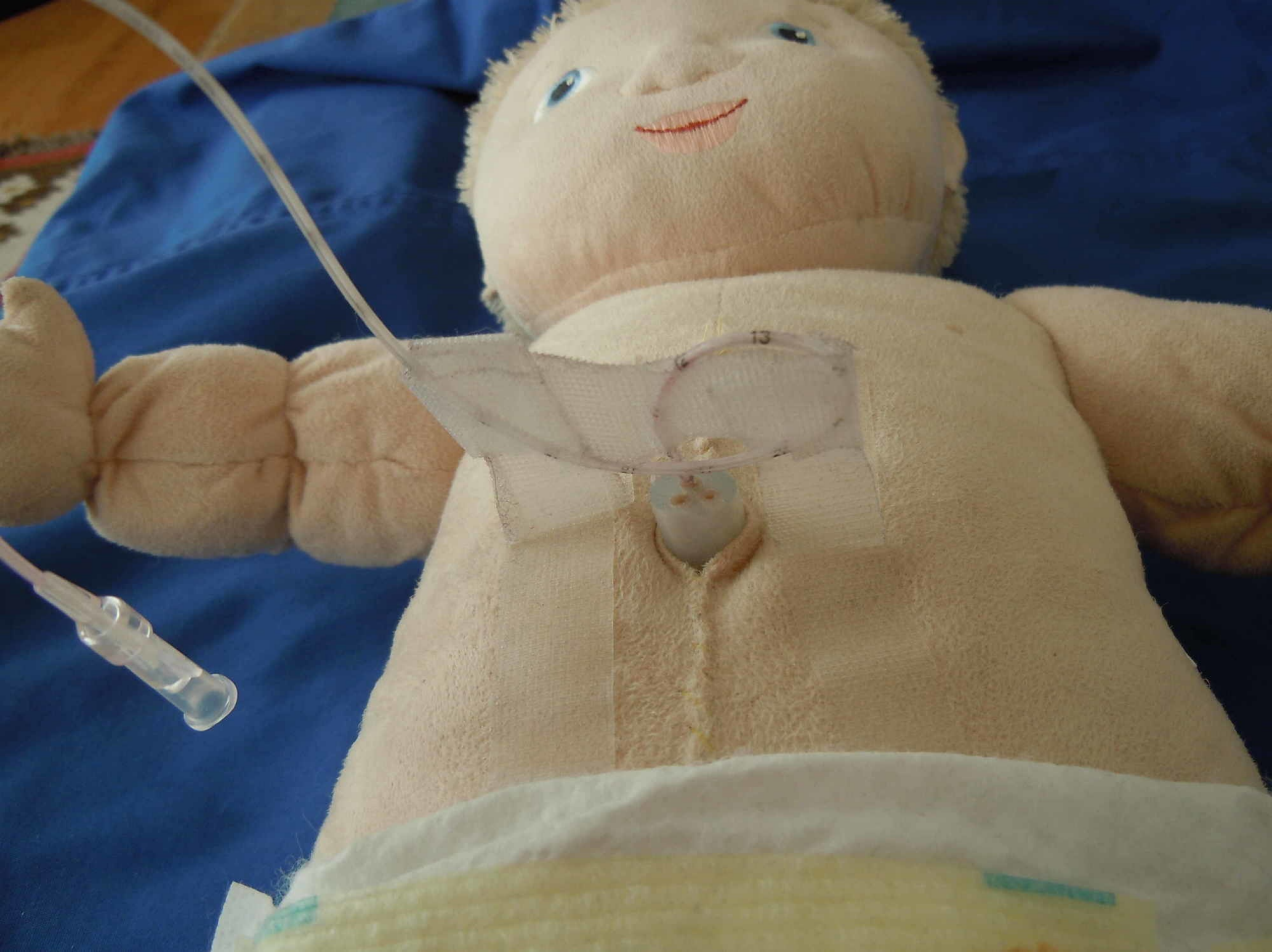
Model with reservoir sewn in place

Another option is to close the fabric without a zipper so the reservoir cannot come out unless it is cut open and re-stitched (Figure 5). This option looks more realistic, but being able to clean the model by adding the zipper is a significant advantage. 

**Figure 6:**
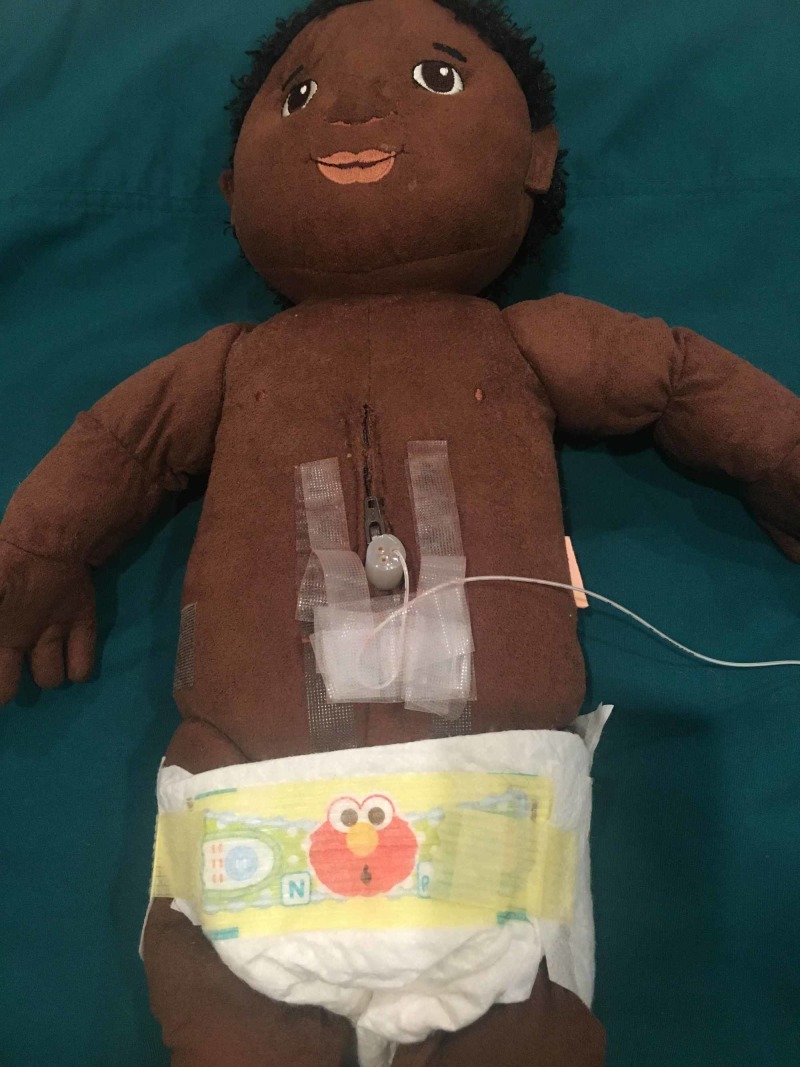
Model with umbilical line secured into reservoir

Umbilical venous access is simulated by placing a 5-French umbilical catheter into the vein in the rubber umbilical stump up to 9 cm. The catheter is secured in place by creating a bridge using adhesive tape affixed to the model’s abdomen (Figure 6). 

**Figure 7:**
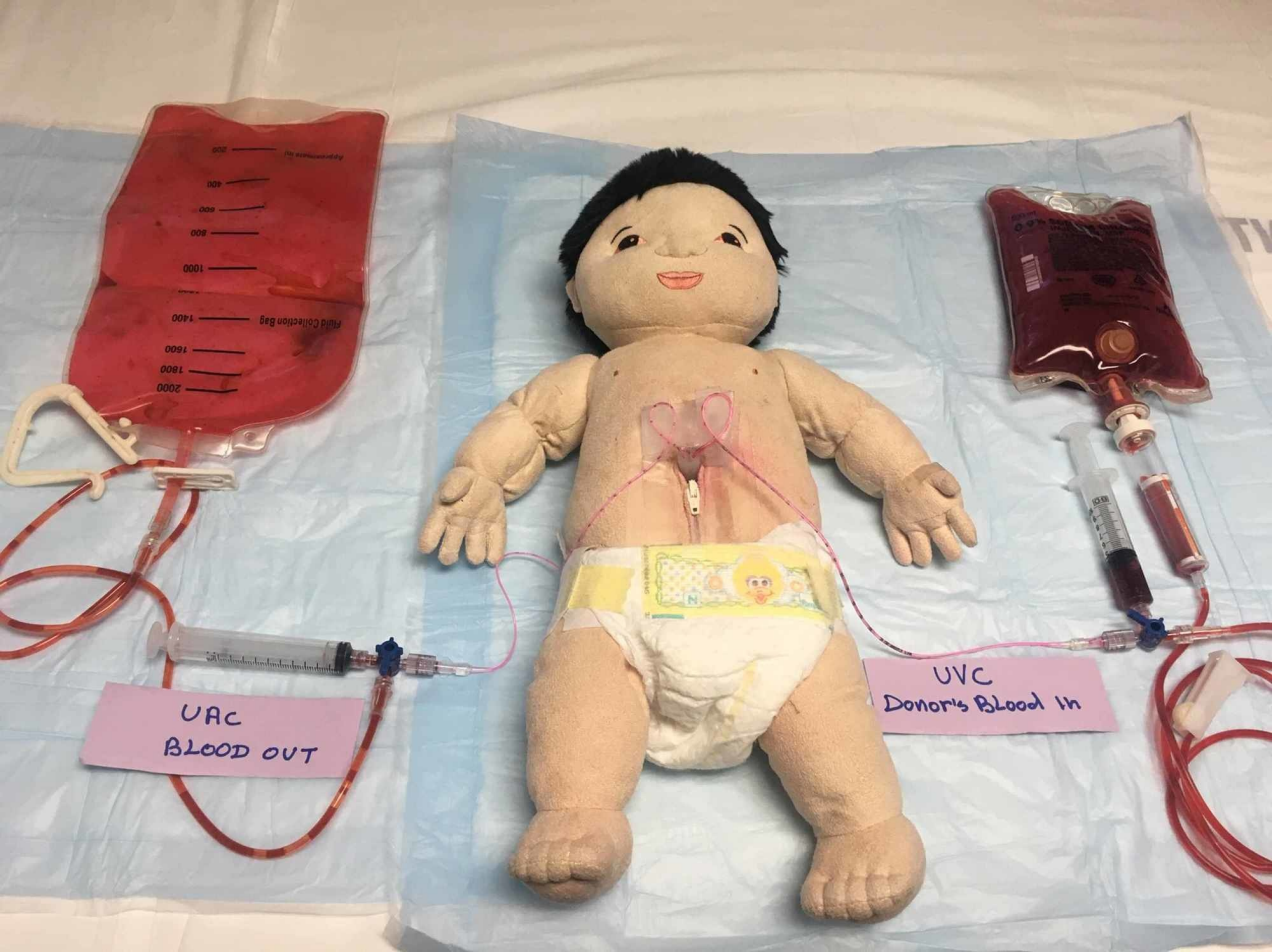
Model for euvolemic exchange with two umbilical catheters

Local preference for securing umbilical lines may be substituted. In order to create a model utilizing both a venous and arterial catheter, a 3.5-French catheter may be inserted into the model and secured as described above or by local practice (Figure 7). This is for practicing a euvolemic exchange transfusion where blood is simultaneously withdrawn and infused. 

Simulated Blood

Simulated blood is created by using a 500 mL bag of normal saline and adding 6 mL of red food coloring and four drops each of green and blue. One tablespoon of cornstarch is mixed in two tablespoons of water to make a slurry. This mixture is injected into the bag of red-dyed normal saline to make the solution turbid, for a more authentic look. The cornstarch can clog a blood filter after using the model several times. This can be remedied by replacing the filter or omitting the cornstarch from the blood mixture, sacrificing the authenticity. 

Single Catheter Exchange

**Figure 8:**
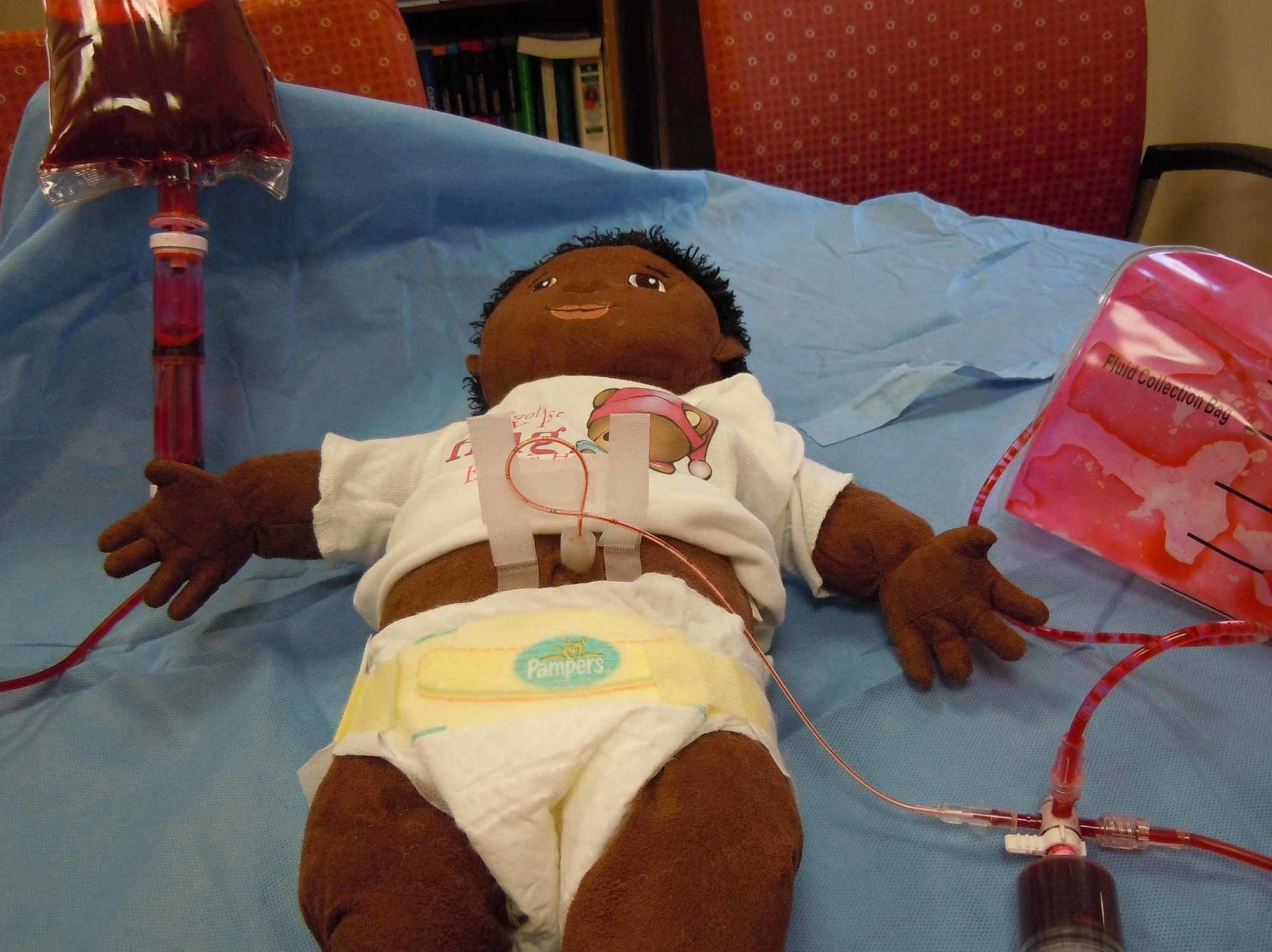
Exchange model with umbilical venous catheter

When using a single catheter, an exchange transfusion kit is assembled per the instructions included (Figure 8). The simulated blood, disposal blood, and umbilical venous catheter are attached to the four-way stopcock as shown. This model requires a single operator to perform the transfusion. 

Two Catheter Exchange (Euvolemic)

Two catheters are inserted using two 3-way stopcocks or a 3-way stopcock and the 4-way stopcock that is included in the kit (Figure 7). The umbilical artery catheter is connected via the 3 or 4-way stopcock to an appropriately sized syringe (10 - 20 ml) and the venesection bag in order to discard the blood. The umbilical venous catheter is attached to the simulated donor blood via a 3 or 4-way stopcock. This model requires two operators to perform the procedure. 

Prefilling the Reservoir

After the model is assembled with one of the options above, the reservoir needs to be filled with 25 - 30 cc of simulated blood prior to starting the procedure. This is accomplished by infusing the simulated blood through the catheter into the reservoir that is inside the manikin.

Learning objectives: DVE transfusion

1. Learn how to and practice setting up for an exchange transfusion 

2. Perform a simulated exchange transfusion using a single catheter or two catheters

3. Anticipate and manage complications from the DVE

Critical action checklist

1. Prepare blood for the DVE transfusion [Appendix, Table 1] [3]

2. Verify adequate supplies are gathered (Blood warming device, exchange transfusion kit, umbilical line insertion supplies

3. Insert umbilical catheter(s) and confirm the position with x-ray 

4. Assemble exchange transfusion kit

5. Send appropriate lab studies before the procedure (complete blood count (CBC), bilirubin (total and direct), serum electrolytes, and ionized calcium)

6. Perform exchange transfusion with appropriate aliquots, recording actions appropriately

7. Monitor for complications during the procedure and after (hypoglycemia, hypocalcemia, hyperkalemia, arrhythmia, air emboli, and acidosis)

8. Monitor length of time for each cycle and monitor patient carefully throughout 

9. Send appropriate blood tests at the end of the procedure (CBC, bilirubin (total and direct), serum electrolytes, and ionized calcium)

Suggested scenario

The full-term infant, birth weight 3.0 kg at 20 hours of life, has a bilirubin level of 17, the rate of rising is 0.7 mg/dL/hour. The blood type is B positive, Coombs positive, and the mother’s blood type is O+. The hematocrit is 28 and the reticulocyte count is 18%. IVIG has been administered at two hours of age and at 14 hours of age; however, the bilirubin is climbing and the hematocrit is dropping.

Calculations for Simulated Scenario [Appendix, Table 1]

1. Formulas to calculate the amount of the blood components to create the desired hematocrit 

2. Double blood volume to be exchanged: 2 X 80 cc/kg X 3 kg = 480 cc plus 30 cc for tubing. 

3. Reconstitution of blood from packed red blood cells (pRBCs) and fresh frozen plasma (FFP) to create a hematocrit of 55%

4. Packed red blood cell (9 pRBC) fraction: volume to be exchanged: (480 cc) x desired hematocrit (55)/hematocrit of pRBCs (60) = 440 cc 

5. FFP volume: double blood volume (480 cc) - pRBC fraction (440 cc) = 40 cc

6. Reconstitution of DVE blood: 440 cc of pRBCs + 40 cc of plasma = 480 cc

Debriefing Plan

1. Review calculations with the trainees, correcting any errors and or misperceptions

2. Individual and group feedback is given after the scenario ends about communication and teamwork

3. Review potential complications and alternative management plans

## Discussion

Since we built these models in 2013, they have been used more than 20 times. Over 50 trainees have practiced the exchange transfusion procedure. The authors have presented these models in national and international simulation meetings (International Pediatric Simulation Symposia and Workshops 2013, Society for Simulation in Healthcare 2014).

To improve the preparation of the subjects, all learners received reading materials prior to the simulation training sessions. We provided feedback during and after the procedures, but we did not videotape these sessions. Learners were surveyed after the simulation sessions, and they expressed higher levels of confidence regarding the procedure compared to pre-training. 

The scenario that is suggested above is a case of hemolysis due to ABO incompatibility and an uncomplicated exchange transfusion. If the stated learning objectives were achieved, then we increased the complexity of the scenario with complications, such as hypocalcemia, hyperglycemia, or thrombocytopenia that appeared during or after the procedure. An additional suggested complication is for the baby to become bradycardic during the procedure. The learner needs to pause the procedure, determine the underlying cause, and then resume with a slower pace once other complications are ruled out (hypocalcemia or hypothermia).

By practicing DVE transfusion, trainees learn to perform this procedure that has become a rare event. The simulated practice takes place in situ or in a remote location. Conducting the simulation off-site allows learners to be more relaxed and focus on learning. 

This model can be easily adapted to teach a dilutional exchange transfusion that is required for polycythemia of the newborn. Instead of using simulated blood as a replacement fluid, one can use normal saline. Another option is to simulate hydrops due to severe anemia. 

Learning the DVE transfusion has cognitive and technical components. Cognitively, trainees need the medical knowledge necessary for performing the DVE procedure, including calculating blood volume, knowing the blood type, calculating the reconstitution of blood components, possible complications, and how to treat them. Technical points include the performance of the DVE with a focus on the technique, including preparation of a sterile field, insertion of the umbilical catheter(s), assembly of the kit, using the blood warmer, performing the exchange, maintaining consistent aliquot size, speed of cycles, and documentation.

The debrief after the scenario is guided by the facilitators, helping the trainees achieve the learning objectives. A group discussion reviewing and reinforcing the learning objectives helps to ensure equal skill and knowledge development. 

## Conclusions

This technical report gives detailed instructions and pictures illustrating how to assemble a task trainer for teaching the performance of an exchange transfusion. This model can be used for any type of exchange transfusion (double volume or partial exchange) and can be made more sophisticated by adding the suggested complications. It is low cost, easy to assemble, reusable, and appears very realistic. Included is a suggested clinical scenario, learning objectives, and critical action checklist. This exchange transfusion task trainer allows providers to learn and practice a rare critical procedure in the field of newborn medicine.

## Appendices

Appendix

**Table 1:** Double Volume Exchange (DVE) Transfusion Calculations

	Checklist
Step 1	Alert blood bank of pending DVE transfusion, calculate appropriate volumes as shown below, and place the blood order
Step 2	Double blood volume to be exchanged: 2 x 80 cc/kg x weight (kg). Assume that a full-term baby has 80 cc/kg of blood
Step 3	Reconstitution of blood from packed red blood cells (pRBCs) and fresh frozen plasma (FFP): A) Calculate the pRBC fraction of the total volume to be exchanged: (double blood volume) x (desired hematocrit) - (pRBC hematocrit) = pRBC volume; B) Calculate the FFP volume: (double blood volume) - (pRBC volume) = plasma volume required to be added to the pRBCs to create the desired hematocrit.
Step 4	Calculate aliquot size: 3 - 5 cc/kg/aliquot
